# Assessment of Change in Enamel Color and Surface Hardness Following the Use of ICON Resin Infiltration and Remineralizing Agent: An In Vitro Study

**DOI:** 10.3390/ma17246030

**Published:** 2024-12-10

**Authors:** Naif Almosa, Khalid Alaman, Fares Alkhudairi, Muhannad Alhaqbani, Mohammed Alshalawi, Rahaf Zawawi

**Affiliations:** 1 Department of Pediatric Dentistry and Orthodontics, College of Dentistry, King Saud University, P.O. Box 60169-38, Riyadh 11545, Saudi Arabia; 2 College of Dentistry, King Saud University, P.O. Box 60169-38, Riyadh 11545, Saudi Arabia; khaled.alaman99@gmail.com (K.A.); farisnfmk@gmail.com (F.A.); md.a.h9000@gmail.com (M.A.); 3 Department of Restorative Dentistry, College of Dentistry, King Saud University, P.O. Box 60169-38, Riyadh 11545, Saudi Arabia; dr.alshalwi@gmail.com; 4 Independent Researcher, P.O. Box 60169-38, Riyadh 11545, Saudi Arabia; rahaf-z@live.com

**Keywords:** demineralization, enamel hardness, remineralizing agent, color change, ICON, CPP-ACFP

## Abstract

This study aimed to evaluate the change in enamel color and surface micro-hardness following the use of resin-infiltration concept material (ICON) and casein phosphopeptide-amorphous calcium fluoride phosphate (CPP-ACFP) remineralizing agent. Fifty-four extracted human third molars were collected and randomly divided into three groups: group A: control with no surface treatment; group B: treated using ICON; and group C: treated using CPP-ACFP. The change in color and micro-hardness of the enamel surface were measured using spectrophotometer and Vickers hardness number, respectively. The measurements were taken at three timelines; baseline (BL), after demineralization (DM), and after surface treatment (TX). The three groups showed no significant differences in enamel color change after demineralization (*p* < 0.05). However, after surface treatment in relation to the baseline, groups B and C had a significant increase in color change compared to the control group (*p* < 0.05), and group B showed a statistically significant increase in enamel color changes compared to group C. Additionally, all groups exhibited a significant reduction in enamel micro-hardness after demineralization in comparison to their baseline (*p* < 0.05). Group C showed a significant increase in micro-hardness after surface treatment compared to groups A and B (*p* < 0.05), while group B showed a significant decrease in enamel micro-hardness compared to groups A and C (*p* < 0.05). These findings suggest that teeth treated with CPP-amorphous calcium fluoride phosphate (CPP-ACFP) show a significant improvement in enamel surface color after demineralization compared to the teeth treated with resin infiltration (ICON) and the non-treated teeth. Additionally, enamel surfaces treated with CPP-ACFP show significant enamel hardness regaining, while resin infiltration (ICON) compromises enamel surface hardness.

## 1. Introduction

Dental enamel is a highly mineralized tissue with a well-packed crystalline structure [1,2]. The composition and arrangement of enamel confers its unique properties, including (A) three-dimensional (3D) aspects of color, achieved via its translucency, opalescence, and ability to transmit light to the underlying dentin, and (B) surface mechanical properties, including surface hardness [2,3].

Orthodontic appliances act as stagnation areas where dental plaque can accumulate, disrupting the typical mineral exchange between the dental enamel and oral fluids, potentially leading to the demineralization of enamel and the development of subsurface porous areas referred to as white spot lesions (WSLs) [4]. These lesions are characterized by their chalky white appearance, resulting from the altered refractive index between the healthy enamel and the surrounding environment [5]. Therefore, WSLs are a common aesthetic concern after orthodontic treatment [6].

The color and 3D aspects of enamel are influenced by intrinsic factors (composition and properties) as well as extrinsic factors, such as diet, non-cavitated caries lesions, and dental bleaching [2]. The concentration of minerals in the enamel directly affects its color and aesthetic [7]. The 3D color of teeth and esthetic restoration can be measured by a spectrophotometer employing the Commission International de l’Eclariage (CIE) system [8]. This system utilizes three color values (L scale for lightness, a scale for red vs. green, and b scale for yellow vs. blue) and their differences to numerically represent the degree of color change that is perceivable by humans [9].

Surface hardness refers to the mechanical resilience of a surface against penetration and deformation [10,11]. Changes in enamel surface hardness can lead to surface degradation, mineral loss, and the development of carious lesions, and the reverse is also true [12,13,14].

Various treatment modalities have been proposed to enhance the aesthetic quality of WSLs, ranging from non-invasive to surgical options [15]. A resin-infiltration approach such as resin-infiltration concept material (ICON) (i.e., one of the most common resin-infiltration materials) approach relies on acid etch of the lesion surface and infiltration of a low-viscosity resin into the inter-crystalline spaces of hypo-mineralized enamel [16]. This approach has shown promising results in improving WSL appearance, with stable outcomes lasting for 6 months [7]. Resin infiltration (ICON) was found to significantly increase the micro-hardness of the demineralized enamel [17].

Another non-invasive approach uses remineralizing agents that minimize the formation of WSLs and regain lost minerals. Casein phosphopeptide-amorphous calcium phosphate (CPP-ACP) is a recently introduced agent that maintains super-saturation of calcium and phosphate ions, thus modulating the bioavailability of calcium phosphate levels and finally leading to an increase in remineralization [18]. CPP-ACP is an effective treatment option that not only promotes remineralization and reduces lesion surface area but also helps restore the aesthetics and function of teeth affected by WSLs [7,19]. Zankalouny et al. found that CCP-ACP improves demineralized enamel micro-hardness, but to a lesser extent than resin infiltration (ICON) [17].

Fluoride addition to CPP-ACP (CPP-ACFP) potentiates its ions binding ability at different pH levels. This leads to the formation of acid-resistant fluorapatite, which has a more potent remineralizing effect on the tooth surface than CPP-ACP alone [20,21,22]. A study evaluated the effect of resin infiltration (ICON) and remineralizing agents (fluoride and CPP-ACP) on remineralization and color stability and found that remineralization potential was highest for CCP-ACP and lowest for ICON. Despite this, ICON provided the highest color improvement with the lowest color variation [23].

To the best of our knowledge, there has been no study comparing the effect of resin infiltration (ICON) and CPP-ACFP on the change in color and micro-hardness of demineralized enamel in a single setting. Therefore, this study aimed to assess the change in enamel color and surface micro-hardness following the use of ICON resin infiltration and CPP-ACFP remineralizing agent on artificially demineralized enamel surfaces. The null hypothesis is that there will be no change in enamel color and surface micro-hardness following the use of ICON resin infiltration and CPP-ACFP on these demineralized enamel surfaces. The alternative hypothesis is that there will be a change in enamel color and surface micro-hardness following the application of ICON resin infiltration and CPP-ACFP.

## 2. Materials and Methods

This study was approved by the Institutional Review Board (IRB) at King Saud University, College of Medicine (Project No. E-22-8167, 22 October 2023) and the College of Dentistry Research Center at King Saud University (Registration No. IR 0477).

### 2.1. Sample Size Calculation

G*Power (3.1.9.4, Franz Faul, Kiel, Germany) was used for sample size calculation at alpha = 0.05 and effect size 0.65, with power = 0.85. A total sample size of 54 specimens was sufficient, with 18 specimens for each group.

### 2.2. Specimens Preparation and Distribution

Fifty-four extracted human third molar teeth were obtained, cleaned with pumice, and stored in 10% buffered formalin. The roots were sectioned from the crowns using a diamond disc underwater cooling technique (IsoMet! Low-Speed Saw, Buehler, Lake Bluff, IL, USA). The crowns were then mounted in a chemical-cure acrylic resin in polyvinyl chloride rings and flattened using 280-, 400-, 600-, 800-, 1200-, and 4000-grit silicon carbide grinding papers.

The specimens were examined under a digital microscope (Model KH-7700, Hirox, Tokyo, Japan) to ensure no residual resin on the teeth surfaces and to exclude teeth with cracks or defects in the enamel structure.

Specimens were randomly assigned into 3 groups with 18 teeth for each (group A, control with no surface treatment; group B, treated using resin infiltration (ICON); and group C, treated using CPP-ACFP).

### 2.3. Artificial Demineralization

All specimens underwent artificial demineralization by immersing them in an acidic solution for 14 days containing 2.2 mM CaCI_2_, 2.2 mM KH_2_PO_4_, and 0.38 mM acetic acid, and adjusted to pH of 4.5 [24]. The specimens were then cleaned and stored in distilled water.

### 2.4. Surface Treatment

**Figure 1:**
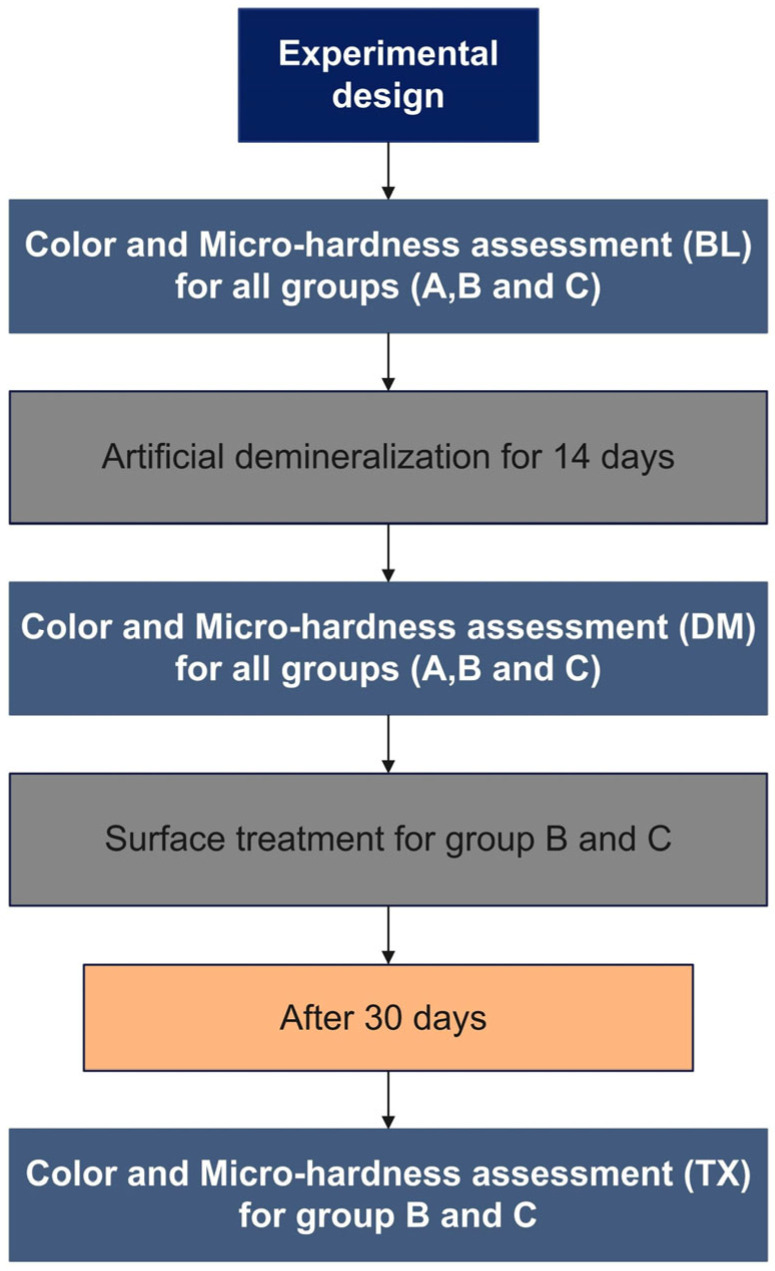
Experimental design (BL: Baseline, DM: After demineralization, and TX: After surface treatment A: Control, B: ICON, and C: CPP-ACFP).

The teeth were treated according to the assigned groups (Figure 1) described in the section below:

**Group A**

Control with no surface treatment.

**Group B**

Resin-infiltration kit (ICON, DMG, Hamburg, Germany) was used and applied following the manufacturer’s instructions. The buccal surfaces were etched using ICON etchant (15% hydrochloric acid) for 2 min. Then, rinsed off for 30 s and air dried for 10 s. ICON-Dry (99% ethanol) was applied for 30 s, followed by air drying for 10 s. Subsequently, ICON-Infiltrant resin was applied to the areas of demineralized enamel and left by a sponge applicator for 3 min. The excess resin was removed by a cotton roll, followed by an LED light-curing unit (Elipar S10, 3M ESPE, St. Paul, MN, USA) for 40 s.

**Group C**

CPP-amorphous calcium fluoride phosphate (CPP-ACFP) was applied for 30 min on the areas of demineralized buccal enamel surface, and the excess was removed with gauze [25]. Then, they were rinsed off using distilled water and stored in artificial saliva (2.38 g Na_2_HPO_4_, 0.19 g KH_2_PO_4_, and 8 g NaCl per liter of distilled water adjusted with phosphoric acid to pH 7.2) [26]. Specimens were kept in an incubator at 37 °C ± 0.5 °C until the next day and the process was repeated for 30 days at the same time while changing the artificial saliva daily [25].

### 2.5. Color Change Assessment

The color change was assessed using a spectrophotometer (LabScan XE, HunterLab, Reston, VA, USA) with a black background at different timelines, baseline-before demineralization (BL), after demineralization (DM), and after surface treatment (TX). A marker was used on the side of each resin block during color measurement to ensure consistent specimen positioning.

The Commission International de l’Eclariage (CIE) L*a*b* values were used to obtain color measurements:

- a* indicates the degree of green or red color.
- b* indicates the degree of blue or yellow color.
- L* indicates the lightness (0 dark, 100 light).

The entire color shift (ΔE) was computed using the mean ΔL*, Δa*, and Δb* values of each specimen at each timeline [∆E_1 (BL/DM)_, ∆E_2 (DM/TX)_, and ∆E_net (BL/DM_DM/TX)_], using the following formula [17]:

ΔE = (ΔL2 + Δa2 + Δb2) 1/2.

Since ∆E is a subtraction of 2 values, ∆E_1 (BL/DM)_ indicates the color change in the specimen after demineralization compared to its baseline. ∆E_2 (DM/TX)_ reflects the color change in the specimen after treatment in relation to the state after demineralization. Finally, ∆E_net (BL/DM_DM/TX)_ signifies the overall color change after treatment compared to the baseline. A (ΔE) difference of >3.7 units is considered a clinical indicator for color change. Hence, (ΔE) of ≤3.7 is considered acceptable for color change [27].

### 2.6. Micro-Hardness Assessment

All specimens were assessed for micro-hardness using a micro-Vickers hardness tester (Innovatest Micro-Vickers Hardness Testers 240, Maastricht, The Netherlands) with a 200 g load for 15 s dwell time at the determined timelines (BL, DM, and TX). Five readings were averaged to obtain the mean Vicker’s hardness number (VHN) [28].

**Statistical Analysis**

The data were entered into MS Excel (2010 version) and analyzed using SPSS (version 26, IBM, Armonk, NY, USA) at a level of significance of *p* ≤ 0.05. Descriptive statistics (mean and standard deviation) were presented. For inferential statistics (testing hypothesis), normality and equality of variance were tested. One-way analysis was performed followed by Tukey’s test for multiple comparisons of mean values.

## 3. Results

### 3.1. Color Change

Normality and equality of variance were satisfied. Descriptive statistics showed that after surface treatment the highest lightness (∆L) color change was for group B with increased lightness in relation to after demineralization (∆L2 _(DM/TX)_ = 80.22 ± 73), which was reduced in relation to baseline (∆L _(BL/TX)_ = 43.27 ± 45.1) (Table 1). The lowest ∆a color change was for group C after surface treatment in relation to after demineralization (∆a2 _(DM/TX)_ = 0.07 ± 0.1), which was increased in relation to the baseline (∆a _(BL/TX)_ = 0.84 ± 2.7) (Table 1). After surface treatment, the highest ∆b color change was for group B in relation to the baseline (∆b _(BL/TX)_ = 12.70 ± 8.7) (Table 1).

**Figure 2:**
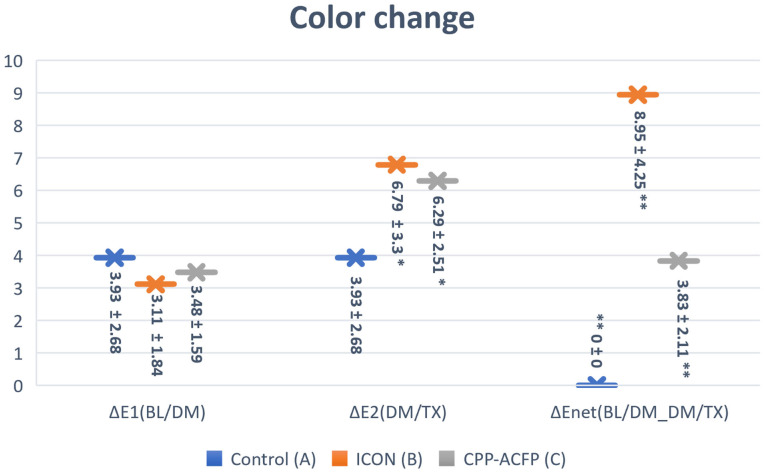
Bar chart showing color change (AE) means values at different times (BL: baseline, DM: after demineralization, and TX: after surface treatment. Error bars represent the ±SD. * level of significance *p* < 0.05, ** level of significance *p* < 0.01. (∆E1 (BL/DM) represents the color change in the specimen after demineralization in relation to their baseline, ∆E2 (DM/TX) represents the color change in the specimen after treatment in relation to after demineralization, and ∆Enet (BL/DM_DM/TX) represents the color change after treatment in relation to their bassline).

The lowest color change values were after demineralization (∆E_1 (BL/DM)_ A: 3.93, B:3.11, and C: 3.48), while the highest color change value was for group B (ICON) after surface treatment in relation to its baseline (∆E _Net (BL/DM_DM/TX)_: 8.95). There were no statistically significant differences between the three groups in the aspect of enamel color change after demineralization (*p* = 0.067). After surface treatment, both groups B and C showed a statistically significant color change with increase ∆E_2 (DM/TX)_ in comparison to the control group (∆E_2 (DM/TX)_: 6.79, 6.29, and 3.93, respectively) (*p* < 0.05) (Figure 2). However, there was no significant difference between groups B and C (∆E_2 (DM/TX)_: 6.791 and 6.290, respectively) (*p* > 0.05). After surface treatment in relation to the baseline, both groups B and C showed a statistically significant increase in color change compared to the control group (*p* < 0.05), and group B showed a statistically significant increase in enamel color changes compared to group C (∆E_Net (BL/DM_DM/TX)_: 8.95 and 3.83, respectively) (*p* < 0.05) (Figure 2).

### 3.2. Micro-Hardness

**Figure 3:**
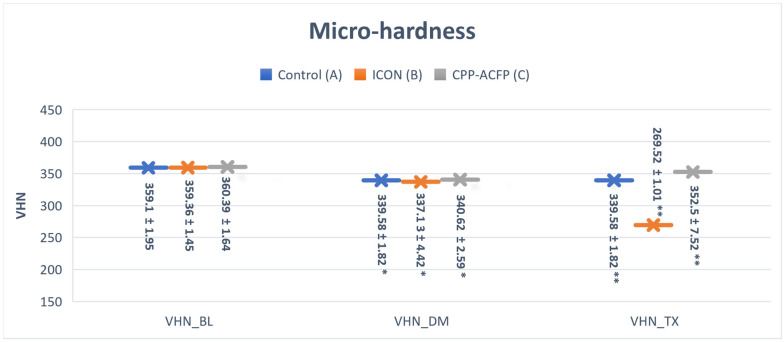
Bar chart showing micro-hardness (VHN: Vicker’s hardness number) means values at different times (BL: baseline, DM: after demineralization, and TX: after surface treatment). Error bars represent the ±SD. * level of significance *p* < 0.05, ** level of significance *p* < 0.01.

Normality and equality of variance were satisfied. The highest micro-hardness mean values were for all groups at the baseline (A: 359.13, B: 359.36, and C: 360.39); these values reduced after demineralization (A: 339.58, B: 337.13, and C: 340.62). The lowest micro-hardness mean value was for group B after surface treatment (269.52). There were no statistically significant micro-hardness differences between the three groups at the baseline (*p* > 0.05). All groups showed a significant surface micro-hardness reduction after demineralization in comparison to their baseline (*p* < 0.05). After surface treatment, group C showed a statistically significant increase in micro-hardness than groups A and B (*p* < 0.05), with group B showing the highest reduction (Figure 3).

## 4. Discussion

The presence of orthodontic appliances often leads to the formation of WSLs, which are areas of demineralized enamel that appear as chalky white spots on the tooth surface [5,6]. Several approaches were introduced to improve the appearance of WSLs with varied impacts on the enamel surface hardness. This study aimed to evaluate the change in enamel color and surface micro-hardness following the use of ICON resin infiltration and CPP-ACFP remineralizing agent on artificially demineralized enamel surfaces.

The color change was assessed using spectrophotometry at different timelines (baseline, after demineralization, and after surface treatment). The results of this study demonstrated that both resin-infiltration (ICON) and CPP-ACFP treatments had increased the lightness ∆L and redness ∆a color change than untreated surfaces. Resin infiltration (ICON) introduced a more yellowish ∆b color change than CPP-ACFP surface treatment and untreated surfaces.

This study showed that CPP-ACFP surface treatment introduced a substantial color change improved than resin-infiltration (ICON) treatment, with a closer value of ΔE to 3.7. These findings are consistent with the study conducted by Cohen et al. reporting that lesions treated with ICON exhibited greater color change difference compared to remineralized lesions, potentially representing an aesthetic disadvantage for the former treatment; however, they utilized a qualitative evaluation approach instead of employing a quantitative readout through spectrophotometry, which could have provided more precise and objective measurements of the color change [8].

Yuan et al. demonstrated that resin infiltration is more effective than NaF or CPP-ACP in achieving aesthetic improvement of white spot lesions [29]. Paris et al. found that resin-infiltration (ICON) treatment was effective in achieving aesthetic improvements when different infiltrating materials were used to evaluate the masking effect of resin infiltration on artificially induced white spot lesions in bovine teeth [30].

Resin infiltration is limited in achieving effective aesthetic improvements due to its reliance on the color blending between the demineralized area and the surrounding enamel. The outcome can be explained by the fact that the enamel surface and most carious lesion bodies were filled with an infiltrating resin matrix. This resulted in only approximating the light reflectivity of the natural enamel surface, masking the chalky spots [31].

Micro-hardness was assessed using Vickers hardness testing at different timelines (baseline, after demineralization, and after surface treatment). All groups showed a decrease in micro-hardness after demineralization. The results of this study demonstrated that both resin-infiltration (ICON) and CPP-ACFP treatments had a significant impact on the enamel hardness.

After surface treatment, the CPP-ACFP group exhibited increased micro-hardness values compared to the control and resin-infiltration groups. Previous laboratory studies have also demonstrated that CPP-ACFP significantly enhances enamel hardness compared to the control group [19,21,32]. This illustrates the beneficial effects of the CPP-ACFP surface treatment on the remineralization of demineralized enamel, which would increase its density through the infiltration of ions of calcium, phosphate, and fluoride into these crystals, leading to an improvement in hardness [33,34].

After surface treatment, the resin-infiltration (ICON) surface treatment led to a significant drop in micro-hardness—having the lowest mean hardness values—compared to CPP-ACFP surface treatment and baseline groups. El Sayed et al. similarly found that ICON surface treatment introduces a lower hardness value than the baseline, but higher than after the demineralization stage [35]. This study’s findings contradict previous observations when resin infiltration was applied [17]. Gurdogan et al. found no significant difference in enamel hardness changes introduced by resin infiltration (ICON) [36].

The dropped hardness findings following the use of resin-infiltration (ICON) surface treatment could be explained by its superficial resin infiltration into enamel porosity and lesion, which introduce weak intermolecular bonding and jeopardize hardness recovery to baseline [37]. Nevertheless, the artificially and deeply introduced lesion and the introduced enamel porosity with the superficial resin infiltration might lead to a lower hardness introduction after surface treatment than baseline and after-demineralization stages.

Park et al. had evaluated the different application time of resin infiltration affect color improvement and found that the higher the penetration time (from 3 to 9 min), the better the color improvement [38]. Since the longer duration enhances the resin infiltration, it is expected to have deeper penetration, which might influence the micro-hardness findings differently. Further studies are required to compare the longer resin infiltration to CPP-ACFP surface treatment effect on color change and its impact on micro-hardness.

The results of this study have clinical implications for the management of white spot lesions following orthodontic treatment. The use of CPP-ACFP satisfactorily improves color aesthetics and enhances enamel hardness, which is crucial for preventing further demineralization and promoting remineralization. On the other hand, the resin-infiltration technique (i.e., ICON) offers limited improvements in color aesthetics and jeopardizes the surface hardness.

The use of extracted human third molars in this study may not fully represent the complex oral environment and individual variations in treatment response that occur in real patients. Conducting further studies on actual patients would yield more clinically relevant findings.

Furthermore, this study relies solely on the surface micro-hardness method to evaluate enamel remineralization. It would have been more advantageous to also employ the transverse microradiography (TMR) method, which is considered the most accurate technique for examining mineral distribution in the deeper layers of enamel. By utilizing TMR, we could have obtained a more comprehensive understanding of the mineral composition of the enamel lesion. The surface micro-hardness test could have been used as an additional tool to assess the physical properties of the enamel lesion. Therefore, for future research, we recommend the transverse microradiography test (TMR).

## 5. Conclusions

Teeth that were treated with CPP-amorphous calcium fluoride phosphate (CPP-ACFP) demonstrated a significant improvement in enamel surface color after demineralization when compared to both resin infiltration (ICON) and untreated teeth. Additionally, there was notable regaining of enamel hardness. In contrast, the resin-infiltration technique (ICON) provided only limited improvements in color while compromising the hardness of the enamel surface. For optimal results in both aesthetics and structural integrity, CPP-ACFP is the superior choice.

## Figures and Tables

**Table 1:** Mean and standard deviation values of ∆L, ∆a, and ∆b at different times ). ∆L1, ∆a1, ∆b1 is the difference between the baseline and demineralization; ∆L2, ∆a2, ∆b2 is the difference after the surface treatment and demineralization; ∆L3, ∆a3, ∆b3, is the difference after surface treatment to baseline. Color change (∆E) means and standard deviation values at different times and mean comparison. For each column, the same superscript small letter indicates no significant difference (*p*-value > 0.05) (ANOVA). For each row, the same superscript capital letter indicates no significant difference (*p*-value > 0.05) (Repeated measurement). (BL: baseline, DM: after demineralization, and TX: after surface treatment).

Group	Mean ± SD								
	∆L_1 (BL/DM)_	∆a_1 (BL/DM)_	∆b_1 (BL/DM)_	∆L_2 (DM/TX)_	∆a_2 (DM/TX)_	∆b_2 (DM/TX)_	∆L_3 (BL/TX)_	∆a_3 (BL/TX)_	∆b_3 (BL/TX)_
**Control (A)**	20.84 ± 34.6	0.22 ± 0.4	1.16 ± 1.9	0 ± 0	0 ± 0	0 ± 0	20.84 ± 34.6	0.22 ± 0.4	1.16 ± 1.9
**ICON (B)**	10.57 ± 13.7	0.04 ± 0	2.25 ± 5	80.22 ± 73	0.46 ± 0.7	16.43 ± 13.3	43.27 ± 45.1	0.43 ± 0.7	12.70 ± 8.7
**CPP-ACFP (C)**	10.49 ± 9	0.9 ± 3.2	3.12 ± 6.1	17.34 ± 20.1	0.07 ± 0.1	1.43 ± 1.7	41.92 ± 34.1	0.84 ± 2.7	2.73 ± 4

## Data Availability

The original contributions presented in this study are included in the article. Further inquiries can be directed to the corresponding author.
